# Developing and validating a scale to measure perceived barriers to prosthodontics treatments among partially edentulous patients

**DOI:** 10.3389/froh.2024.1517574

**Published:** 2025-01-10

**Authors:** Rayan Sharka, Majd Alghamdi, Eman Dustakir, Mansour Alghamdi

**Affiliations:** ^1^ Oral and Maxillofacial Surgery Department, Faculty of Dental Medicine, Umm Al-Qura University, Makkah, Saudi Arabia; ^2^ Dental Teaching Hospital, Faculty of Dental Medicine, Umm Al-Qura University, Makkah, Saudi Arabia

**Keywords:** perceived barriers, psychometrics, prosthodontics, patient perceptions, dental services

## Abstract

**Background:**

People experiencing tooth loss need dental prostheses to preserve the integrity of their oral structures and replace the missing teeth. Patient-related outcome measures (PROMs) for perceived barriers to prosthodontics treatment are scarce in the literature.

**Aims:**

The aim of this study was to develop a comprehensive scale to identify and measure barriers to prosthodontic treatment as perceived by partially edentulous patients.

**Methods:**

This cross-sectional study was conducted among *N* = 334 partially edentulous adults who seeking prosthodontic treatment. Data collection was carried out in February to September 2024. Exploratory factor analysis was utilized to elucidate the latent factor structure. A six-factor model was validated through confirmatory factor analysis. Reliability was evaluated using Cronbach's alpha. The convergent and discriminant validity of the final scale were assessed.

**Results:**

EFA produced a 21-item scale grouped into six factors that explain 75.78% of the total variance with eigenvalues >1. All items showed acceptable reliability, ranging from 0.807 to 0.935. The first factor pertained to financial constraints; the second factor was concerning lack of knowledge and awareness; the third factor was related to anxiety and fear; the fourth factor related to negative past dental experiences; the fifth factor included issues related to limited accessibility to dental services; and the last factor was concerning insufficient dental guidance. The CFA results indicate an acceptable model fit, with standardized factor loadings spanning from 0.54 to 0.99. The model factors’ convergent and discriminant validity were confirmed.

**Conclusion:**

This study enhances the understanding of barriers to prosthodontic treatment in a Saudi Arabian teaching dental hospital. It introduces a novel scale for further data collection, aiding policymakers and stakeholders in addressing these barriers and improving public oral health. Future studies should validate this scale and explore its applicability in various contexts and populations.

## 1 Introduction

Teeth loss represents a significant oral health issue among the adult population in Saudi Arabia, frequently attributable to dental caries, periodontal diseases, infections, and orthodontic extractions (1–3). A 2020 study in Riyadh reported that around 56.5% of Saudi adults aged 35–74 had lost at least one tooth (2). In addition, another study in Dammam reported that most (47.58%) adult patients who attended prosthetic dental clinics came with either partial or complete loss of posterior teeth (1). Moreover, a previous study conducted in the eastern region of Saudi Arabia found that 46.7% of the study sample required prosthodontic treatment (4). Tooth loss can have a significant impact on the general health and well-being of individuals (5), with the absence of teeth impairing the ability to chew and process food effectively, potentially leading to significant dietary modifications and subsequent nutritional deficiencies (6, 7). In addition, the absence of dental tissues decreases the stimulation of an alveolar ridge that subsequently leads to bone resorption and loss (8). This osteoclastic activity may cause adjacent teeth to erupt into the edentulous site, thereby compromising dental positioning and occlusion (9). Since teeth are also integral to phonetic articulation, their absence can compromise speech clarity (10, 11). The psychosocial impact of tooth loss is also considerable, often diminishing self-esteem and altering self-perception due to changes in appearance (12, 13). Consequently, patients suffering from tooth loss need dental prostheses to maintain the integrity of existing oral structures and replace the missing dentition (12). Such treatments not only aim to restore aesthetic and functional dimensions but also address a myriad of health-related and quality-of-life concerns (13, 14).

In this current era of modern dentistry, several dental prosthetic options exist to address the issue of tooth loss including removable partial dentures, fixed dental prostheses, and dental implants. The selection of dental prostheses is based on the patient, their preferences, and suggestions from the dental professional (15, 16). However, a range of interrelated barriers including economic, socio-cultural, resources, and dental care system issues also impede the uptake of prosthodontic treatment (17–21). Geographical disparities further exacerbate this issue, as individuals residing in rural or underserved regions often encounter a dearth of specialized dental professionals and facilities (22). Also, socio-cultural impediments, including inadequate oral health literacy, entrenched cultural beliefs, language barriers, and dental anxiety, further complicate the pursuit of prosthodontic care (22, 23). Systemic inefficiencies, such as convoluted administrative processes and restricted appointment availability, may also contribute to the overall inaccessibility of these essential services (24, 25).

Patient-related outcome measures (PROMs) can encompass perceived barriers (26). These measures capture patients’ views on their health and the healthcare services they receive, including barriers they face in treatment or self-management (26). The current body of literature is conspicuously deficient in comprehensive and systematic investigations of perceived barriers to prosthodontics treatment. This gap underscores the need for more expansive and nuanced research endeavours to thoroughly comprehend the intricate challenges faced by patients intending prosthodontic interventions. Taking these into consideration, this study aims to develop a comprehensive scale to identify and measure barriers to prosthodontic treatment as perceived by partially edentulous patients. The findings will provide the literature with a comprehensive tool to assist policymakers in addressing and prioritizing their plans and strategies for prosthodontic dental care.

## 2 Materials and methods

### 2.1 Ethical consideration

The study protocol received approval from Umm Al-Qura University's ethical review committee (No. HAPO-02-K-012-2023-09-1705).

### 2.2 Study setting and subjects

All patients provided written informed permission for participation. The study was carried out from February to September 2024 at the Umm Al-Qura University dental teaching hospital in Saudi Arabia. The hospital offers comprehensive dental treatment services across all dental specialties. Additionally, the hospital provides dental care to patients from diverse backgrounds and nationalities, ensuring inclusive and accessible services for the community.

The study included patients who were at least 18 years old, able to complete consent forms, and had at least one partially edentulous space that was untreated for more than 3 months and without dental prosthetic restorations. The study excluded patients under 18 years old who were unable to communicate their experiences or read the questionnaire, as well as those who had previously received dental prostheses.

**Figure 1:**
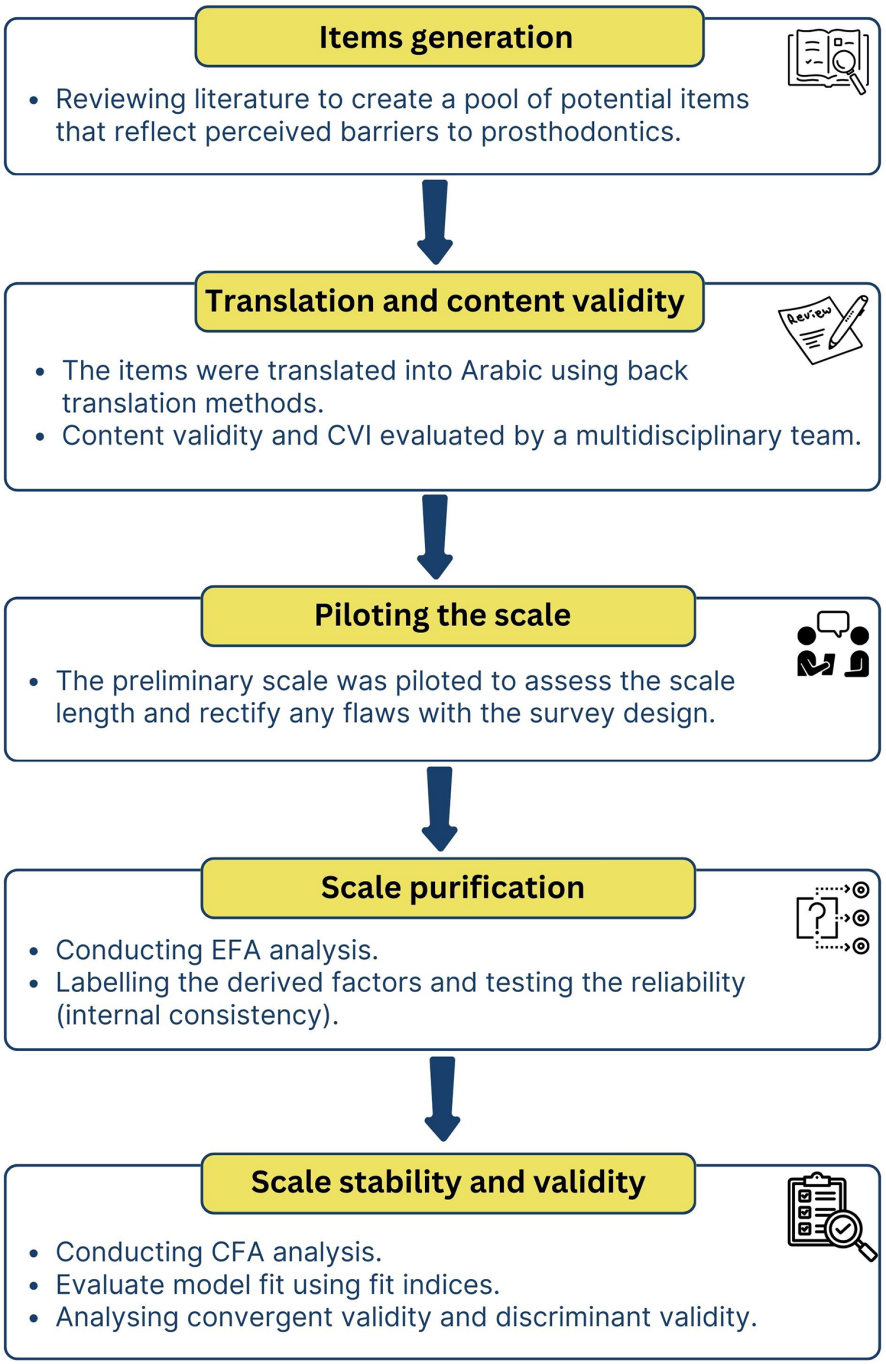
A flow chart that details the main steps taken in this study to create and validate the scale.

The Raosoft online sample calculator was used to ascertain the sample size for the purpose of the study. The required sample size was 278 patients, given a 5% margin of error, a 95% confidence interval, a response distribution of 50%, and a population of around 1,000. To accommodate for incomplete data or missing answers, the sample size was inflated by 20%, resulting in 334 patients being asked to participate in the study. Figure 1 presents a flow chart that details the main steps taken in this study to create and validate the scale.

### 2.3 Development of the scale

#### 2.3.1 Items generation from literature review

We initiated our study with an extensive literature review to identify potential barriers that patients might associate with not receiving dental prostheses (1, 2, 12, 17–19, 27, 28). The goal was to develop item pools by identifying items from the literature that seemed to reflect these perceived barriers. From the literature review, we identified a total of 23 potential items.

#### 2.3.2 Content validity and piloting the scale

The generated items from literature review was translated into Arabic language followed the back translation methodologies outlined in the previous studies (12, 29). Then, a committee from the author's dentistry school, including six faculty members from the prosthodontics and public health departments, was tasked with evaluating the scale for content and face validity in accordance with the content validity procedure outlined in the preceding studies (30). The team performed an independent evaluation to determine the suitability of the items. Only items with a content validity index (I-CVI) value of 0.8 or above were included in the study (30). Two items were removed as it did not met this criteria and achieved an adequate degree of content validity see Supplementary Appendix 1. Specifically, the two items concerning communication barriers were excluded because the experts did not consider them significant barriers. The potential justification could be that, within the context of our study population, language differences are not prevalent to significantly impact dental care.

The definitive version included 21 items of the scale was tested with 15 patients excluded from the main study to identify and rectify any flaws with the survey design, including unclear or difficult questions, prior to the main administration of the scale. This preliminary testing phase ensures that the questions are clear and relevant to patients, thereby enhancing the quality and reliability of the data collected.

#### 2.3.3 Administering the final scale to participants

In the initial part of the questionnaire, participants were asked to provide responses to demographic questions, including gender and age. The subsequent section required participants to assess their level of agreement with 21 statements related to their reasons for not opting for prosthodontic treatment to replace missing teeth. Responses were measured on a five-point Likert scale, where 1 represented strong disagreement and 5 represented strong agreement.

### 2.4 Data management and analysis plan

The statistical analysis was conducted using IBM SPSS statistics for Windows (Version 29.0. Armonk, NY IBM Corp). To examine categorical variables like gender and age, descriptive statistics were calculated. This analysis included frequency distributions and percentage calculations.

The preliminary psychometric evaluation was conducted using exploratory factor analysis (EFA). The principal component extraction method was implemented to explore latent structures within the assessed items and retain maximal variance within the dataset. Subsequently, a varimax rotation technique was employed to enhance the interpretability and clarity of the data structure (31, 32). To fulfil the assumptions of EFA, the normality of the data was assessed using the histograms and Q-Q plots. The Kaiser-Meyer-Olkin (KMO) test was used to assess the sampling adequacy. A threshold of 0.80 or higher is deemed satisfactory. Additionally, Bartlett's Test of Sphericity was employed to evaluate whether the intercorrelations among the variables in the dataset are sufficient to warrant the application of factor analysis. A significant result (*p* < 0.05) indicates that the correlation matrix significantly deviates from an identity matrix, thereby affirming the appropriateness of factor analysis for the data (33). The determination of the number of factors to extract in the EFA was undertaken using a multifaceted approach. Initially, eigenvalues exceeding 1 were considered, adhering to Kaiser's criterion. Furthermore, a scree plot was scrutinized to discern the “elbow” point, where the plot begins to plateau, thereby indicating the optimal number of factors. Lastly, the cumulative variance explained by the factors was evaluated, ensuring that the retained factors encapsulated a substantial proportion of the total variance, typically targeting a threshold exceeding 60%. Additionally, each item must have communalities exceeding 0.50 to be retained in the analysis (34). Cronbach's alpha was employed to evaluate the internal consistency of items corresponding to each extracted factor. A threshold of 0.7 or above is generally regarded as indicative of satisfactory reliability (34).

Confirmatory Factor Analysis (CFA) with the Maximum Likelihood Estimation method is employed subsequent to EFA to validate and corroborate the factor structure elucidated during the exploratory phase. While EFA serves an exploratory function to identify potential factors, CFA rigorously tests and confirms the hypothesized measurement model, ensuring its robustness and reliability. The CFA was estimated using the Amos software (Version 29; IBM Corp., Chicago, IL, USA). The adequacy of the model fit was evaluated model fit indicators, including *χ*^2^/df ≤ 3.0, Comparative Fit Index (CFI), Bentler-Bonett normed fit index (NFI), Tucker Lewis Index (TLI), and the Root Mean Square Error of Approximation (RMSEA) (31, 33, 35). CFI, NFI, and TLI over 0.90, as well as values of RMSEA below 0.08 also indicated a satisfactory model fit (36, 37). These fit indices provide a quantitative evaluation of the congruence between the hypothesized model and the observed data, facilitating the identification of any discrepancies and informing necessary model adjustments. These indices offer critical diagnostic insights for the assessment and enhancement of theoretical models.

Convergent validity of the factors is confirmed when the following criteria are met, the average variance extracted (AVE) for each factor must exceed 0.50, the path loadings for each item within each factor should be greater than 0.5, and composite reliability (CR) should be at least 0.60 (12). Discriminant validity is confirmed if the square root of the AVE for each factor is greater than the correlations between factors (12).

## 3 Results

### 3.1 Demographic characteristics of participants

We invited 334 patients to complete questionnaires, and 307 agreed to participate, yielding an overall response rate of 91.9%. Of the 307 participants, 118 (38.4%) were male, and 189 (61.6%) were female. Approximately 119 (38.8%) were aged 18–39 years, while 188 (61.2%) were 40 years old and above. Additionally, 235 (76.5%) were Saudi, and 72 (23.5%) were non-Saudi.

### 3.2 Exploratory factor analysis (EFA)

The KMO coefficient was found to be 0.835, indicating that the sample size was sufficient. Bartlett's test for sphericity produced statistically significant results (*X*^2^ (210) = 4387.252, *p* < 0.001), confirming that the assumptions for EFA were met.

**Figure 2:**
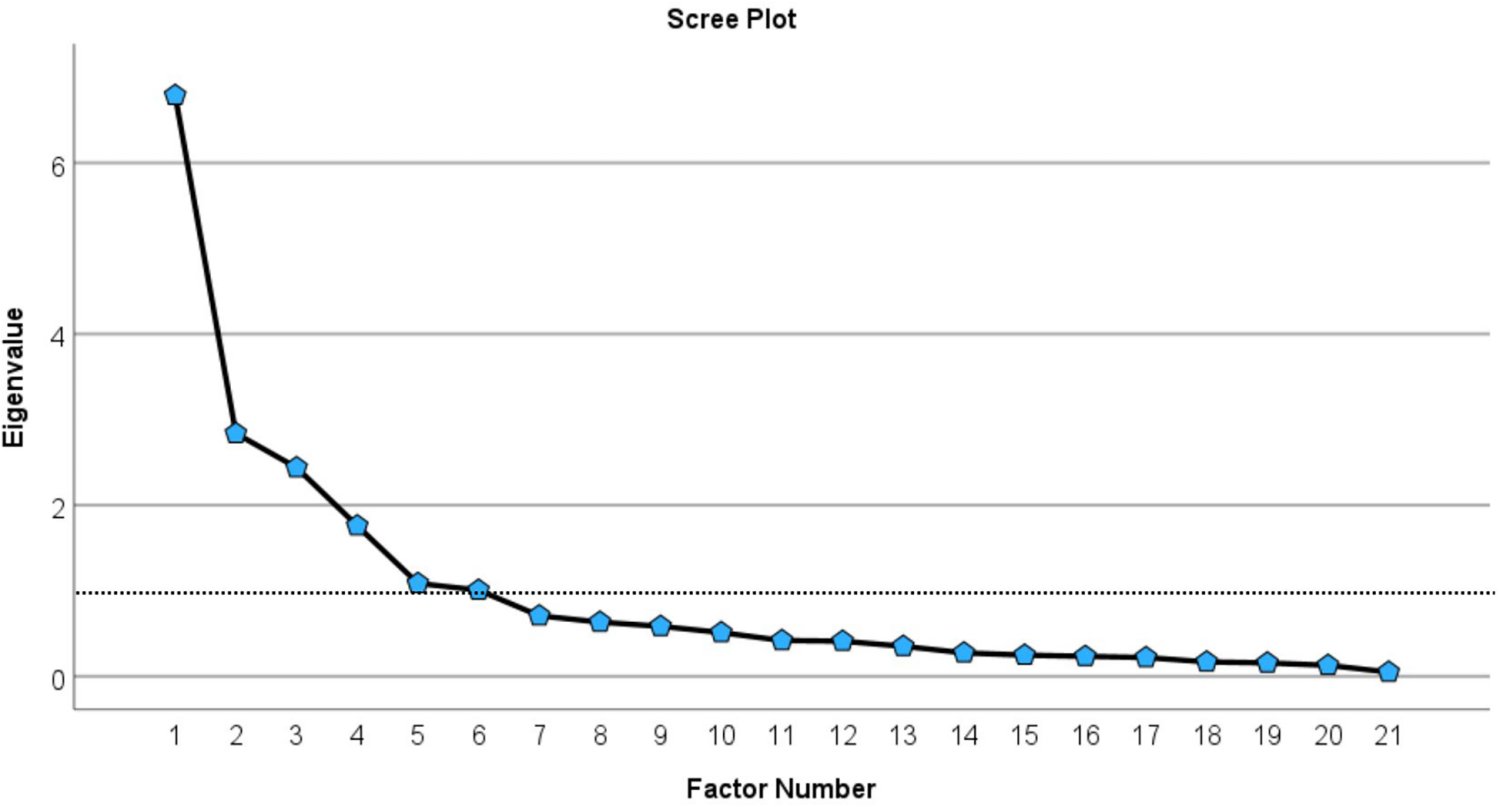
A scree plot analysis of exploratory factor analysis.

The EFA revealed six distinct factors, encompassing a total of 21 items, each characterized by eigenvalues exceeding one. These factors collectively accounted for 75.78% of the total variance. The six extracted factors were further validated through scree plot analysis, as illustrated in Figure 2. The rotated component matrix, detailed in Table 1, presents significant loadings for each item. Moreover, all items exhibited high communalities (*h*^2^) above 0.5, signifying that each item was well-represented within the factor model see Table 1.

**Table 1:** The 21 items identified from exploratory factor analysis using principal component extraction displayed in the rotated component matrix from highest to lowest variance.

Perceived barriers items	Factors						
	1	2	3	4	5	6	*h* ^2^
Item 8: The fees for dental restoration are higher than my financial capability.	0.886						0.836
Item 9: The prosthodontic treatment is expensive.	0.848						0.780
Item 10: My financial capability could not cover the fees for dental prostheses.	0.885						0.829
Item 11: I do not have medical insurance to cover the cost of dental prostheses.	0.801						0.682
Item 3: I have not asked my dentist about how to restore my missing teeth with a dental prosthesis.		0.530					0.500
Item 4: I'm unaware of the need to restore the empty space after losing or extracting teeth.		0.796					0.679
Item 5: I do not know the treatment modalities to restore my missing teeth.		0.826					0.723
Item 6: I have no idea about dental prostheses.		0.803					0.700
Item 7: I'm misinformed about prosthodontic treatment.		0.697					0.557
Item 12: I'm afraid of dentists.			0.947				0.927
Item 13: I'm afraid of dental clinics.			0.949				0.941
Item 14: I get anxious during dental procedures.			0.860				0.806
Item 15: I had unpleasant experiences with previous dentists.				0.842			0.817
Item 16: My past dentist was not good enough.				0.872			0.827
Item 17: My past experiences with dental care center services were not good.				0.837			0.813
Item 18: There are few dental clinics that provide prosthodontic treatments.					0.875		0.853
Item 19: There are few dentists who provide prosthodontic treatments.					0.860		0.867
Item 20: The waiting list in the public dental hospital was long.					0.459		0.509
Item 21: Accessibility to dental clinics is difficult.					0.450		0.512
Item 1: I have never received advice from a dentist on the possibility of having my tooth restored.						0.872	0.908
Item 2: I have never received advice from a dentist on dental prosthesis options for restoring teeth after extraction.						0.853	0.879
% of variance	16.469	14.861	12.877	12.599	10.474	8.505	
Cronbach alpha (*α*)	0.909	0.831	0.935	0.891	0.807	0.909	

Extraction Method: Principal Component Analysis; Rotation Method: Varimax with Kaiser Normalization; Rotation converged in 6 iterations; *h*^2^: Communality; blank cell: Items with loadings <0.35.

### 3.3 Factors reliability and labelling

The Cronbach's alpha (*α*) scores for the six factors ranged from 0.807 to 0.935, indicating excellent internal consistency. This range indicates that the items within each factor reliably measure the same underlying factors, as detailed in Table 1. Following a meticulous examination of the items, each factor was described and appropriately labelled to enhance interpretability see Table 2.

**Table 2:** The extracted factors with descriptions and labels.

Factors	Description	Labeling
Factor I	It refers to the economic barriers that prevent or restrict individuals from receiving the necessary prosthodontic care. These constraints encompass insufficient personal financial resources, inadequate insurance coverage, and the substantial costs associated with prosthodontic procedures and materials.	Financial constraints to prosthodontics treatment
Factor II	It denotes to a deficiency in understanding and recognizing the necessity, benefits, and options available for restoring missing teeth. This condition may arise from limited access to pertinent information, poor education, or negligence of patients for dental care.	Lack of knowledge and awareness
Factor III	It describes the feelings of stress, worry, or fear that some individuals experience when thinking about or undergoing dental procedures.	Anxiety and fear
Factor IV	It refers to any distressing or traumatic events that a person has encountered during previous dental visits.	Negative past dental experiences
Factor V	It refers to barriers that hinder individuals from obtaining necessary prosthodontic care, including a shortage of prosthodontic specialists and dental clinics, as well as geographical limitations.	Limited accessibility to dental services
Factor VI	It refers to a lack of adequate professional instructions, or support provided to patients regarding their prosthodontic care.	Insufficient dental guidance

### 3.4 Confirmatory factor analysis (CFA)

**Figure 3:**
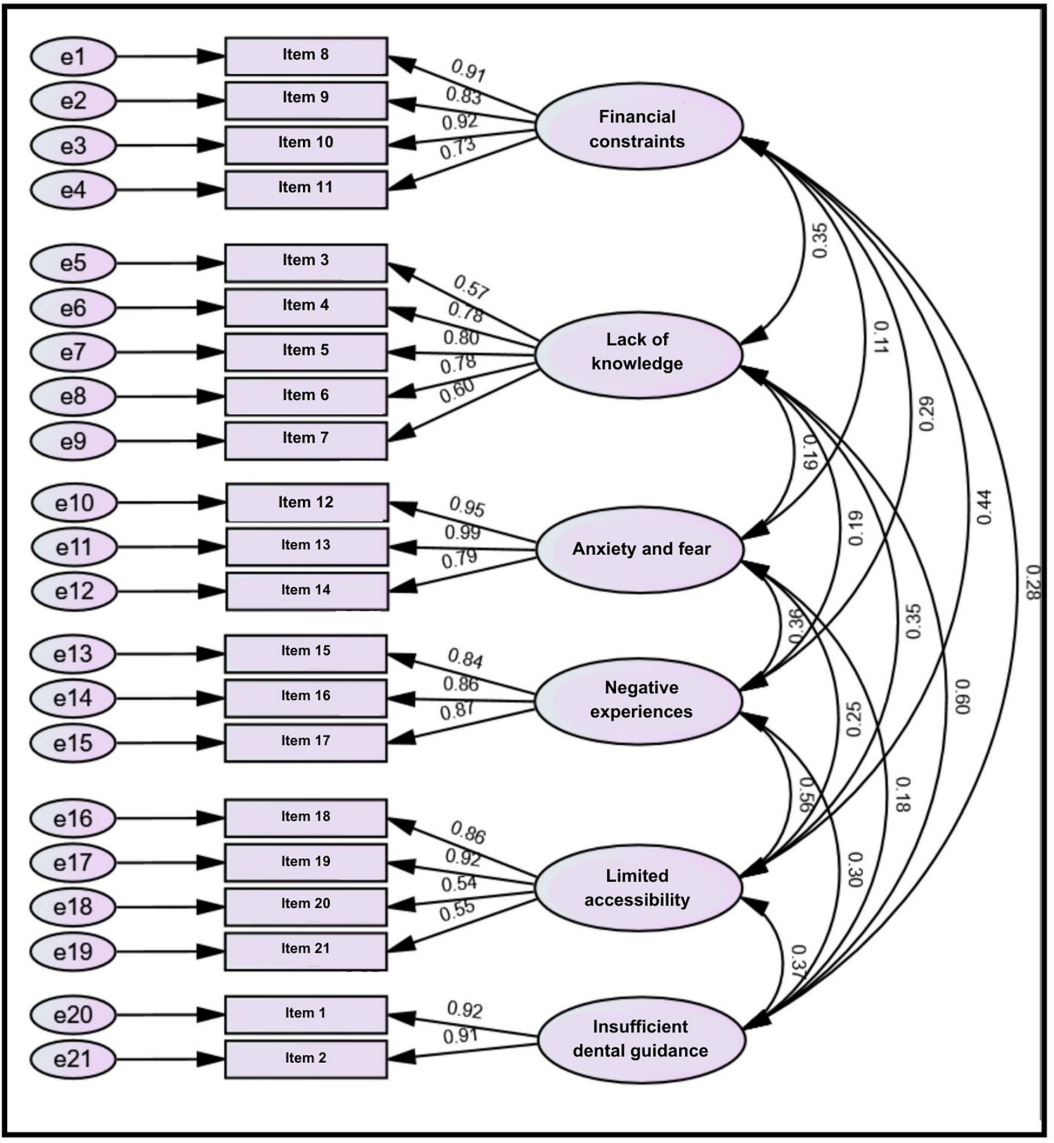
Standardized estimates of the confirmatory factor analysis.

The estimated model had a satisfactory level of fit. The ratio of *χ*^2^/*df* was 2.088, and the model fit indices were as follows: NFI = 0.919, CFI = 0.956, TLI = 0.947, and RMSEA = 0.060. The CFA estimate indicates that the measurement model demonstrates a satisfactory level of fit. Additionally, the standardized path loadings for each item were greater than 0.5, indicating a strong association between the items and their respective factors see Figure 3.

### 3.5 Convergent and discriminant validity

The AVE values for the six factors exceeded 0.5, demonstrating adequate convergent validity see Table 3. Furthermore, the CR values were above 0.6, indicating a robust level of reliability in the measurement model see Table 3. The model met all the requirements for convergent validity. Discriminant validity was also confirmed in this study, as the square root values of AVE for the six factors were higher than the correlation estimates between the factors see Table 4.

**Table 3:** Summarized results of descriptive measures of factors and convergent validity.

Factors	(CR)^a^	AVE^b^	Mean	Std. deviation
Financial constraints to prosthodontics treatment	0.912	0.723	3.991	1.150
Lack of knowledge and awareness	0.836	0.509	2.974	1.110
Anxiety and fear	0.939	0.839	2.703	1.413
Negative past dental experiences	0.891	0.732	2.925	1.301
Limited accessibility to dental services	0.819	0.545	3.442	1.059
Insufficient dental guidance	0.910	0.835	2.824	1.367

^a^ CR, composite reliability.

^b^ AVE, average variance extracted.

**Table 4:** Correlation matrix of factors.

Factors	1	2	3	4	5	6
1. Financial constraints to prosthodontics treatment	**0** **.** **850**	0.329	0.142	0.275	0.498	0.259
2. Lack of knowledge and awareness	0.329	**0**.**714**	0.173	0.168	0.343	0.538
3. Anxiety and fear	0.142	0.173	0.**916**	0.370	0.269	0.170
4. Negative past dental experiences	0.275	0.168	0.370	**0**.**856**	0.542	0.274
5. Limited accessibility to dental services	0.498	0.343	0.269	0.542	**0**.**738**	0.337
6. Insufficient dental guidance	0.259	0.538	0.170	0.274	0.337	**0**.**914**

All correlations significant at *p* < 0.05.

Average variance extracted (AVE) displayed in the diagonal in bold.

## 4 Discussion

A scale of measurement for barriers to prosthodontic treatment as perceived by adult patients was developed to identify and quantify various barriers that adults experience in seeking prosthodontic care. This tool should provide valuable insight into patient experiences that may pinpoint areas of improvement in dental services for better patient outcomes and accessible prosthodontic care.

There were six perceived barriers to prosthodontic treatment among partially edentulous patients. The first barrier was financial constraints to prosthodontics treatment. Previous studies found that the financial implications of prosthodontic treatment significantly influence patients’ decision-making processes (13, 38) considering the high cost of prosthodontic treatment and the patient's financial status. The high costs associated with prosthodontic procedures, such as dental implants, crowns, and bridges, often serve as a substantial barrier to accessing or accepting necessary care (27). Financial constraints can lead to the postponement or complete avoidance of prosthodontic treatment, thereby exacerbating oral health issues over time.

Evidence showed that financial restraints were always the main reason for not restoring missing teeth or affecting the treatment plan options (12, 27, 39). Patients from lower socioeconomic backgrounds are more susceptible to these financial obstacles, since they may lack the necessary means to finance these kinds of treatments (22). Even when individuals have health insurance, dental coverage sometimes fails to sufficiently cover the full expenses of prosthodontic procedures (40). This financial strain forces many people to choose short-term financial security above long-term oral health, resulting in untreated dental conditions that could have been effectively managed or prevented with timely intervention. Consequently, conditions that could have been managed or prevented with routine care become more complex and costly to treat.

Interestingly, the lack of knowledge and awareness emerged as the second factor and explained 14.861% of the total variance. In this context, the lack of awareness may include insufficient knowledge about how to restore missing teeth and the options available for treatment. Patients may not recognize the importance of addressing missing teeth when they lack adequate information about the benefits and necessity of prosthodontic interventions (27). This gap in understanding can lead to a reluctance to pursue treatment, as patients might not fully grasp the potential consequences of untreated dental issues (41).

Previous studies demonstrated that a lack of awareness regarding the importance of prosthodontics is among the primary reasons for the failure to restore missing teeth (27, 42). Moreover, the absence of awareness about the various prosthodontic options available can result in patients feeling overwhelmed or uncertain about their choices. Without proper guidance from dental professionals, patients may not be aware of the advancements in prosthodontics that can provide effective and aesthetically pleasing solutions (41). This lack of information can contribute to a sense of apprehension or fear regarding the procedures, further diminishing their willingness to consider prosthodontic treatments.

Anxiety and fear represent significant psychological barriers that can markedly influence a patient's willingness to seek prosthodontic treatment. Moreover, they can profoundly impact individuals’ lives, frequently resulting in the avoidance of dental visits, deterioration in dental health, and a reduced quality of life associated with oral health (43). These states may stem from a myriad of sources including prior adverse dental experiences, apprehension regarding pain, and overarching dental anxiety (44).

Previous studies demonstrated that younger individuals typically manifest elevated levels of dental anxiety relative to their older counterparts due to their limited exposure to dental procedures and an amplified fear of the unknown (44, 45). In contrast, older adults generally report diminished levels of dental anxiety, likely attributable to their greater familiarity with dental treatments and an enhanced capacity to manage anxiety-provoking situations (45). Additionally, females are more prone to dental anxiety than males, possibly due to heightened pain sensitivity and a stronger tendency to recall negative dental experiences (46, 47). In the context of prosthodontic treatment, which encompasses the restoration and replacement of teeth, it can present significant challenges for individuals experiencing elevated levels of anxiety and fear as these procedures typically necessitate numerous appointments and may be viewed as intrusive, heightening patients’ anxiety.

The fourth factor was negative past dental experiences including the patient's past unpleasant experiences with the dentist and the dental clinic services. These experiences often leave lasting psychological scars, leading to dental anxiety or even phobia (44). Such intense fear can create a strong aversion to future dental visits, making patients reluctant to seek necessary prosthodontic care, even in the presence of pain or significant discomfort.

However, when dental professionals meticulously curate a positive dental appointment experience, it significantly augments patient loyalty and incentivizes the continuation of care with the same professional (48, 49). Furthermore, creating a positive dental experience encourages patients to be more open to accepting suggested treatments, returning for future care, and recommending the same dentist to friends and family (20). This approach not only strengthens the professional standing and practice of the dentist but also significantly improves the overall oral health of the patient (48).

The fifth factor was limited access to dental care services and also included the patient's perceptions of the number of dentists and dental clinics as well as the accessibility to dental services. Patients residing in rural or underserved areas may face considerable challenges in finding dental professionals, particularly specialists in prosthodontics. Consequently, they often experience higher rates of oral health issues and missing teeth, especially among underserved populations (27, 44).

Empirical evidence demonstrated a correlation between edentulism and constrained accessibility to dental care. Inadequate access to dental services frequently leads to higher rates of untreated dental problems, including tooth loss (50, 51). Furthermore, a systematic review highlighted that tooth loss is significantly correlated with diminished oral health-related quality of life (OHRQoL), a condition often exacerbated by restricted access to dental care (52). Also, the swift progress in materials and technologies, coupled with the growing challenges and demands in the current dental care system, particularly due to an aging population with heightened expectations, is placing increasing pressure on prosthodontic services (53).

The final barrier was insufficient dental guidance. Dental professionals play a pivotal role in maintaining oral health and disseminating critical information regarding various dental interventions, including the necessity of prosthodontic treatments. It is incumbent upon dental professionals to elucidate the consequences of untreated tooth loss, such as tooth migration, bone resorption, and changes in occlusion (54) as without proper professional guidance, patients may not fully comprehend the importance of prosthodontic treatment, resulting in a diminished motivation to seek such care.

According to Paterick et al., providing patients with comprehensive education regarding their oral health conditions, available treatment options, and necessary maintenance protocols empowers them to actively engage in their care (55). This empowerment fosters improved adherence to treatment plans, facilitates informed decision-making, and promotes proactive management of their overall health (55). A study conducted in Australia revealed that patients highly valued dentists who demonstrated empathy, respect, and attentiveness to their concerns without assigning blame for their oral health condition. These patients appreciated dental practitioners who clarified current dental problems and preventative measures, conveyed information on maintaining optimal dental hygiene, and continually offered support and education throughout their appointments (56). Thus, the lack of dental advice can create a significant barrier to patient awareness and confidence regarding prosthodontic treatment.

### 4.1 Study implications

A novel model was developed that holds significant potential for enhancing prosthodontic care among the population in Saudi Arabia and provides a comprehensive framework for policymakers and stakeholders to identify and address gaps in current prosthodontic services. The model aims to enhance the accessibility, quality, and efficiency of prosthodontic care by integrating evidence-based practices and patient-centered approaches.

The scale offers empirical data for policymakers to inform the development of targeted health policies and programs. For example, identifying financial barriers can guide resource allocation toward subsidizing prosthodontic treatments or expanding insurance coverage. The model highlights financial constraints as significant barriers that must be addressed, underscoring the need for targeted financial strategies, such as subsidizing costs, increasing funding for public health programs, and implementing insurance reforms to make prosthodontic services more affordable and accessible.

Moreover, the developed scale serves as a practical tool for clinicians to systematically identify and address barriers faced by patients in seeking prosthodontic treatment. Clinicians can use this scale during patient consultations to tailor their communication and treatment plans to better meet individual patient needs, thereby enhancing patient compliance and satisfaction. Additionally, the lack of knowledge and awareness about the necessity of prosthodontics following tooth extraction highlights the increased responsibility of dental professionals to educate their patients. This gap in understanding can lead to delayed or neglected prosthodontic care, adversely affecting oral health and overall well-being. Dental professionals must take proactive steps to inform patients about the benefits of timely prosthodontic interventions, including the prevention of further dental complications and the enhancement of quality of life. Finally, the scale can aid in designing public health campaigns aimed at raising awareness about the importance of prosthodontic care, ultimately leading to improved oral health outcomes at the population level.

### 4.2 Study limitations and future research directions

The conduct of this study in a single-teaching public hospital limits its generalizability to the entire population of Saudi Arabia, therefore multi-center studies across different geographic regions are recommended to improve the external validity and generalizability of future research. Additionally, future studies should incorporate patient perceptions from private clinics to provide a more comprehensive understanding of the barriers as financial issues may not be the predominant barriers. Another study limitation is the lack of specific questions about participants’ income or socioeconomic status as such information may significantly influence the interpretation of the results. Future research should consider incorporating these demographic details to provide a more comprehensive understanding of the construct across different contexts.

Moreover, in this study, both EFA and CFA were performed on the same sample which may introduce bias and increase the risk of overfitting. Future studies should consider splitting the sample into two independent groups: one for EFA and another for CFA. Furthermore, this study used questionnaires as the primary data collection instrument, resulting in limited depth and the potential omission of nuanced patient insights. Future research should consider utilizing qualitative methods, such as interviews and focus groups, to yield more comprehensive and in-depth findings. Finally, future research should incorporate a measurable scale for prosthodontic treatment demands and inequalities, particularly focusing on older adults from underprivileged backgrounds and totally edentate patients.

## 5 Conclusion

This study enhances the understanding of perceived barriers to prosthodontic treatment among a sample from a teaching dental hospital in Saudi Arabia. The developed scale for collecting further data will help policymakers and stakeholders address and mitigate these barriers, ultimately contributing to the improvement of public oral health. Further studies should be conducted to validate this scale and explore its applicability in different contexts and populations.

## Data Availability

The raw data supporting the conclusions of this article will be made available by the authors, without undue reservation.
