# Supplementary material for: Developing and validating a scale to measure perceived barriers to prosthodontics treatments among partially edentulous patients
Source: Front Oral Health. 2025 Jan 10;5:1517574. doi: 10.3389/froh.2024.1517574 (PMC11757889; doi:10.3389/froh.2024.1517574)
Supplement: Supplementary file 1 [file Datasheet1.pdf]

Developing and Validating a Scale to Measure Perceived Barriers to Prosthodontics Treatments

The relevance ratings on the item scale by 6 experts

Item	Expert 1 SA (Prostho)	Expert 2 ER (Prostho)	Expert 3 HA (DPH)	Expert 4 BF (DPH)	pert 5 AT (Prost	Expert 6 GG (DPH)	Experts in agreement	I-CVI	UA
I have never received advice from a dentist on dental prosthesis options for restoring teeth after extraction.	1	1	1	1	1	1	6	**1**	1
I have never received advice from a dentist on the possibility of having my tooth restored.	1	1	1	1	1	1	6	**1**	1
Accessibility to dental clinics is difficult.	1	1	1	1	0	1	5	**0.83**	0
The waiting list in the public dental hospital was long.	0	1	1	1	1	1	5	**0.83**	0
There are few dentists who provide prosthodontic treatments.	1	1	1	1	1	1	6	**1**	1
There are few dental clinics that provide prosthodontic treatments.	1	1	1	1	1	1	6	**1**	1
My past experiences with dental care center services were not good.	1	1	1	1	1	1	6	**1**	1
My past dentist was not good enough.	0	1	1	1	1	1	5	**0.83**	0
I had unpleasant experiences with previous dentists.	1	1	1	1	1	1	6	**1**	1
I get anxious during dental procedures.	1	1	1	1	1	1	6	**1**	1
I'm afraid of dental clinics.	1	1	1	1	1	1	6	**1**	1
I'm afraid of dentists.	1	1	1	1	1	1	6	**1**	1
I'm misinformed about prosthodontic treatment.	1	1	1	1	1	1	6	**1**	1
I have no idea about dental prostheses.	1	1	1	1	1	1	6	**1**	1
I find it difficult to communicate my dental concerns to my dentist due to language differences.	0	0	0	0	1	1	2	**0.33**	0
I'm unaware of the need to restore the empty space after losing or extracting teeth.	1	1	1	1	1	1	6	**1**	1
I do not know the treatment modalities to restore my missing teeth.	0	1	1	1	1	1	5	**0.83**	0
I do not have medical insurance to cover the cost of dental prostheses.	1	1	1	1	1	0	5	**0.83**	0
I am not confident in my ability to communicate effectively with the dental staff.	0	0	0	0	1	1	2	**0.33**	0
My financial capability could not cover the fees for dental prostheses.	1	1	1	1	1	1	6	**1**	1
The prosthodontic treatment is expensive.	1	1	1	1	1	1	6	**1**	1
The fees for dental restoration are higher than my financial capability.	1	1	1	1	1	1	6	**1**	1
I have not asked my dentist about how to restore my missing teeth with a dental prosthesis.	1	1	1	1	1	1	6	**1**	1
							S-CVI/Ave	5.43	
Proportion relevance	0.78	0.91	0.91	0.91	0.96	0.96	S-CVI/UA		0.70
	The average of proportion relevance scores across all experts						0.91		

Based on the above calculation, we can conclude that I-CVI, and S-CVI/Ave meet satisfactory level, and thus the scale of questionnaire has achieved satisfactory level of content validity.
